# Ranibizumab for Visual Impairment due to Diabetic Macular Edema: Real-World Evidence in the Italian Population (PRIDE Study)

**DOI:** 10.1155/2015/324841

**Published:** 2015-07-29

**Authors:** Ugo Menchini, Francesco Bandello, Vincenzo De Angelis, Federico Ricci, Luigi Bonavia, Francesco Viola, Elisa Muscianisi, Massimo Nicolò

**Affiliations:** ^1^Department of Specialized Surgical Sciences, Eye Clinic, University of Florence, 50121 Florence, Italy; ^2^Department of Ophthalmology, University Vita-Salute Scientific Institute, San Raffaele, Via Olgettina, 20132 Milan, Italy; ^3^A.O.R.N.A. Cardarelli, 80131 Naples, Italy; ^4^UOSD Patologie Retiniche, Fondazione PTV, Università di Roma Tor Vergata, Viale Oxford 81, 00133 Rome, Italy; ^5^A.O. Polo Universitario Luigi Sacco, Università degli Studi, Grassi 74, 20156 Milan, Italy; ^6^U.O. Oculistica, Fondazione IRCCS Cà Granda Ospedale Maggiore Policlinico, Università di Milano, 20122 Milan, Italy; ^7^Novartis Farma S.p.A, 21040 Origgio, Italy; ^8^Clinica Oculistica, DiNOGMI, Università di Genova, IRCCS Azienda Ospedaliera Universitaria “San Martino-IST”, 16132 Genova, Italy; ^9^Fondazione per la Macula Onlus, 16121 Genova, Italy

## Abstract

*Purpose*. An expanded access program (PRIDE study) in Italy to provide ranibizumab 0.5 mg to diabetic macular edema (DME) patients, prior to reimbursement. *Methods*. Open-label, prospective, phase IIIb study. Majority of patients were not treatment-naïve before enrollment. Patients received ranibizumab as per the EU label (2011). Safety was assessed by incidences of ocular/systemic adverse events (AEs) and serious AEs (SAEs) and efficacy in terms of visual acuity (VA) change from baseline (decimal score or Snellen (20/value)). *Results*. Overall, 515 patients (83.5%) completed the study. In unilateral/bilateral patients, commonly observed AEs were cardiac disorders (1.3%/1.3%) and nervous system disorders (1.3%/1.1%); SAEs were reported in 4.5%/4.8% of patients. Acute renal failure, lung carcinoma, and cardiac arrest were the causes of death in one unilateral and two bilateral patients. Ranibizumab improved/maintained VA (Snellen (20/value)/decimal scores) in both unilateral (up to −16.7/1.5) and bilateral patients (up to −23.6/1.2) at Month 5, with a mean of 4.15 and 4.40 injections, respectively. Overall, no difference was observed in the VA outcomes and treatment exposure between unilateral/bilateral patients. *Conclusions*. The PRIDE study provided early ranibizumab access to >600 Italian patients. Ranibizumab was well-tolerated and improved/maintained VA in 40.2%–68.8% patients, with no differences in case of unilateral or bilateral pathology. The study is registered with EudraCT.

## 1. Introduction

Diabetic macular edema (DME) is one of the leading causes of visual impairment in the working-age population in developed countries [1]. Clinical trials data from Europe and the United States indicate that, of the total diabetic population, 7%–12% suffer from DME and 1%–3% have visual impairment due to DME [2]. The World Health Organization (WHO) estimates that the number of Europeans with diabetes is expected to increase from 33 million (4 million in Italy) in 2000 to 48 million (5 million in Italy) in 2030, with a likely corresponding increase in the prevalence of visual impairment due to DME [3]. Therefore, in addition to systemic control of diabetes, identifying treatment strategies to effectively manage patients with DME is imperative. In Italy, there is currently no data available on the prevalence and incidence of legal blindness (residual vision 1/20 (20/400 feet, 6/120 meters) or lower for both the eyes) in patients with diabetes [4]. Epidemiological data from Italy show that at least 30% of patients with diabetes suffer from retinopathy and that every year 1% of patients with diabetes are affected by the most severe forms of retinopathy [5].

In the past, treatment options for DME were limited to laser photocoagulation, triamcinolone intravitreal injections, and vitrectomy [6–8]. Most of these target the prevention of vision loss and result in limited visual acuity (VA) improvements, while causing safety concerns such as foveal burns, visual field defects, retinal fibrosis, and laser scars [9].

Of the antivascular endothelial growth factors (VEGF) that are currently being used or investigated for ophthalmic conditions, ranibizumab (Lucentis; Novartis Pharma AG, Basel, Switzerland, and Genentech Inc., South San Francisco, CA) was the first VEGF inhibitor approved by the Committee for Medicinal Products for Human Use (CHMP), for the treatment of DME. Ranibizumab is a humanized monoclonal antibody Fab fragment (without the Fc portion) specifically designed for ocular use. It binds to VEGF-A with high affinity and inhibits all the isoforms of VEGF-A [10]. Data for treatment of DME with ranibizumab are based on prospective phases II and III randomized clinical studies, including READ-2, RESOLVE, RISE & RIDE, RESTORE, RESTORE extension, DRCR.net (Protocol I), REVEAL, and LRT (NCT00444600) [11–20]. Ranibizumab 0.5 mg pro re nata (PRN) was identified as the new standard of care following the conclusion of the RESOLVE [16] and RESTORE [11] studies. At 12 months, these studies showed that ranibizumab pooled group (RESOLVE; 0.3 mg–0.6 mg and 0.5 mg–1.0 mg) and ranibizumab 0.5 mg pro re nata (RESTORE) provided superior VA gains compared with controls (RESOLVE, 10.3 versus −1.4 with sham control, *p* < 0.0001; RESTORE 6.8 versus 0.9 with laser control, *p* < 0.0001) [21]. Long-term data from the 2- and 3-year RESTORE extension [17, 18] and DRCR.net (Protocol I) [13, 14] studies, respectively, demonstrated that individualized ranibizumab 0.5 mg dosing maintained the initial BCVA improvement observed at Year 1 through Years 2 and 3, with lower number of injections. In order to provide early access to ranibizumab for patients with no therapeutic alternative other than laser, Novartis Farma S.p.A initiated an expanded access program (EAP) in Italy (the PRIDE study) in 2011. Ranibizumab was administered to the patients according to the then approved label until reimbursement for DME indication and was available at the referral center according to the Hospital Formulary System (Prontuario Terapeutico Ospedaliero (PTO)/Prontuario Terapeutico Ospedaliero Regionale (PTOR)) requirements. This EAP provided the opportunity to evaluate the safety and efficacy of ranibizumab in Italian patients with DME akin to the real-world/routine clinical practice. Additionally, most of the DME patients (over 90%) included in the study were not treatment-naïve prior to study enrollment. The study also analyzed the efficacy in patients with very poor vision (best-corrected visual acuity (BCVA) <20/320), who are typically excluded from clinical trials. Here, we present the 18-month results from the PRIDE study.

## 2. Materials and Methods

### 2.1. Study Design

PRIDE was a phase IIIb, open-label EAP study with a maximum of 18 months of follow-up period, conducted in 33 centers in Italy from November 7, 2011, to October 8, 2013, in patients with visual impairment due to DME (unilateral and bilateral). Informed consent was obtained from each patient in writing at screening, before any data were collected or procedure was performed. The study was conducted in accordance with the Declaration of Helsinki and approval was obtained from the Independent Ethics Committee or Institutional Review Board in all the centers. The study is registered with https://eudract.ema.europa.eu/ (2011-002731-26).

### 2.2. Patients

The study enrolled 617 patients, ≥18 years of age with either Type I or Type II diabetes mellitus according to the American Diabetes Association or WHO guidelines and visual impairment due to focal/diffuse DME in at least one eye for which no suitable therapeutic alternatives existed. The study included patients with both unilateral DME and bilateral DME; if both eyes were eligible, the one with the worst VA, as assessed at visit 1, was selected for treatment in patients with bilateral DME unless, based on medical reasons, the investigator deemed the other eye more appropriate for treatment (similar to the RESTORE study) [11]. Patients were excluded if they had a history of uveitis in either eye, uncontrolled glaucoma in either eye (intraocular pressure (IOP) >30 mmHg on medication or according to the investigator's judgment), evidence of either vitreomacular traction (in either eye) or active proliferative diabetic retinopathy (study eye), usage of intravitreal antiangiogenic drugs within 1 month before enrollment, pan retinal laser in the study eye within 6 months before enrollment, focal/grid laser in the study eye within 3 months before enrollment, or hypertension uncontrolled by medication.

### 2.3. Study Objectives

The objective of the study was to provide an early access to ranibizumab for patients with visual impairment due to DME for whom no suitable therapeutic alternatives existed (i.e., existing therapies, e.g., laser photocoagulation, have failed or were not indicated) and to generate safety data of ranibizumab 0.5 mg treatment (administered according to the European Union Summary of Product Characteristics (EU SmPC) label, 2011) in an Italian population that resembles future clinical practice.

In our study, we analyzed the dataset seeking a difference between unilateral versus bilateral pathology in patients with DME; however, only unilateral treatment (one study eye) was allowed.

### 2.4. Treatment

Enrolled patients received intravitreal ranibizumab 0.5 mg injections as per the then approved ranibizumab EU SmPC label, 2011. Initially, three consecutive monthly doses of ranibizumab 0.5 mg were administered until the maximum VA was achieved. The treatment was suspended upon stabilization of vision, that is, when there was no further improvement in BCVA attributable to ranibizumab treatment for two consecutive visits. Patients were monitored monthly, and if a decrease in VA due to disease activity was observed, monthly ranibizumab treatment was resumed until VA was stable for 3 consecutive monthly assessments. All patients were eligible to receive laser treatment at a minimum interval of 90 days according to the Early Treatment Diabetic Retinopathy Study (ETDRS) guidelines at the investigators' discretion (recorded as concomitant medication). If both ranibizumab and laser were given on the same day, then ranibizumab was administered at least 30 minutes after laser treatment.

### 2.5. Assessments

#### 2.5.1. Treatment Exposure

The proportion of patients receiving an injection at each visit, as well as the total number of injections administered from baseline to the end of the study (i.e., maximum until Month 18), and the number of visits, during which injections could be administered, were computed and described by the means of usual descriptive statistics. In addition, the ratio between the total number of injections performed and the number of visits during which ranibizumab could be administered was determined. The number of patients with at least one study drug interruption was also calculated. Time to first retreatment, defined as the time elapsed (in days) from the last performed injection when VA reached stability to the date of first injection following first treatment interruption, was computed.

#### 2.5.2. Safety

Safety was assessed by the incidence of ocular and systemic adverse events (AEs) and serious adverse events (SAEs), standard ophthalmic examinations and IOP measurements, and so forth. The incidence of AEs and SAEs based on the standardized Medical Dictionary for Regulatory Activities was summarized by the primary system organ class (SOC) and preferred term.

#### 2.5.3. Efficacy

The total VA was assessed at all scheduled visits using the Snellen VA charts (i.e., evaluations expressed in Snellen 20/value fraction and as decimal scores). Efficacy outcomes are presented in terms of total VA change from baseline (the Snellen scale and decimal scores) and in terms of proportion of patients with improvement, worsening, or no change in total VA at every study visit in the first 6 months of the treatment. Moreover, the efficacy was also assessed in the subgroup of patients (i) who were monocular or had a BCVA score of ≤24 letters (approximately <20/320 Snellen equivalent) in the fellow eye and (ii) with at least 6 months of follow-up.

### 2.6. Statistical Analysis

The safety analysis was conducted on the safety set. It consisted of all patients who received at least one dose of ranibizumab and had undergone at least one postbaseline safety assessment. The efficacy analysis was conducted on the per protocol population as well as in the following subgroups: patients with at least 6 months of follow-up and in patients who were monocular or with VA <20/320 Snellen in the fellow eye. Changes of total VA versus baseline were calculated in categorical terms. (i) Using the Snellen scale, “improvement” was defined as a decrease in the total VA fraction denominator at the considered visit versus baseline, “no change” was defined as no difference in the total VA fraction denominator at the considered visit versus baseline, and “worsening” was defined as an increase in the total VA fraction denominator at the considered visit versus baseline. (ii) Using the decimal score, “improvement” was defined as an increase of the considered visit fraction versus baseline, “no change” was defined as no difference in the considered visit fraction versus baseline, and “worsening” was defined as a decrease of the considered visit fraction versus baseline. Data were summarized using frequency counts and percentage for categorical variables, mean, median, least square (LS) mean, standard deviation (SD), standard error (SE), and min-max values for continuous variables. A Wilcoxon signed rank sum test to evaluate (with descriptive meanings only) the statistical significance of VA change at 6 months versus baseline was performed for both unilateral and bilateral patients with DME.

## 3. Results

### 3.1. Patient Disposition and Baseline Characteristics

A total of 617 patients were enrolled into the study, of which 612 received at least one dose of the study treatment (safety set). The baseline demographic and disease characteristics were generally well-balanced among the unilateral (*n* = 157) and bilateral (*n* = 455) patients with DME (Table 1).

**Figure 1:**
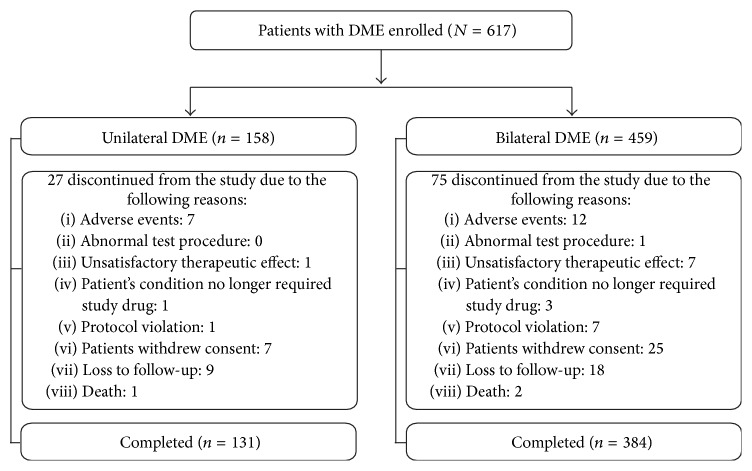
Patient disposition.

A total of 515 (83.5%) patients completed the study (Figure 1). Overall, 102 of the 617 enrolled patients discontinued the study before the program's closure. The main reasons for discontinuation were consent withdrawal (32, 5.2%), patients lost to follow-up (27, 4.4%), and AEs (19, 3.1%). Notably, 56 (9.1%) of the total enrolled patients had ocular disorders in the study eye that could confound the interpretation of results, compromise VA, or require medical or surgical intervention during the study period. The ocular disorders in such patients included cataract, retinal vascular occlusion, retinal detachment, macular hole, or choroidal neovascularization of any cause (e.g., age-related macular degeneration, ocular histoplasmosis, or pathologic myopia). In addition, 18 (3%) patients received laser treatment in the study eye during the study (reported as protocol deviations). About 143 (31.4%) bilateral DME patients and 41 (26.1%) unilateral DME patients had received bevacizumab treatment (for nonocular conditions); 7 (1.5%) bilateral DME patients and 1 (0.6%) unilateral DME patient had received ranibizumab treatment prior to study enrollment while 1 (0.22%) patient with bilateral DME had received dexamethasone treatment prior to study enrollment.

### 3.2. Treatment Exposure

#### 3.2.1. Average Number of Injections

On average, the study eye of unilateral and bilateral patients with DME received a mean (median) of 4.15 (4.00) and 4.40 (4.00) ranibizumab injections, respectively (Table 2), with 7 visits on average over the first 6 months of follow-up. Most patients received 2 to 6 injections. A majority of patients received injections in the range of 2 to 5 (interquartile range: 3–5) for unilateral DME (77.0%) and in the range of 3 to 6 (interquartile range: 3–6) injections for bilateral DME (70.8%). The ratio of number of injections to the number of visits was, on average, 0.62 ± 0.18 for unilateral and 0.65 ± 0.19 for bilateral DME eyes.

In the subgroup of patients with at least 6 months of follow-up, the median number of injections was 4 (interquartile range: 3–5) in patients with unilateral DME and 5 (interquartile range: 4-5) in patients with bilateral DME.

#### 3.2.2. Treatment Interruption

Overall, 86.6% (*n* = 136/157) of the unilateral and 87.7% (*n* = 399/455) of the bilateral patients achieved stable ocular condition at least once during the study that could last for more than one visit and hence suspended ranibizumab treatment. The number of visits during which injections were not performed for these patients was, on an average, 3.22 ± 2.45 (unilateral) and 2.83 ± 2.27 (bilateral; ranges for both: 1–12) over the 18-month follow-up period.

In the subgroup of patients with at least 6 months of follow-up, the proportion of patients who discontinued treatment due to stable ocular condition at any visit from baseline to Month 6 were 82.4% in the unilateral group and 75.3% in the bilateral group.

#### 3.2.3. Retreatment

Retreatment was defined as reinitiation of injections after the interruption of ranibizumab treatment. A total of 28.7% (*n* = 39/136) of the unilateral and 26.8% (*n* = 107/399) of the bilateral patients resumed ranibizumab treatment at least once after having reached VA stability. Time to first retreatment (LS mean ± SE) was, on an average, 160.37 ± 7.16 (unilateral) and 175.17 ± 5.93 (bilateral) days. The differences between unilateral and bilateral DME were not statistically significant as determined by the log-rank test.

In the subgroup of patients with at least 6 months of follow-up, time to retreatment (LS mean ± SE) was 102.28 ± 2.79 (unilateral) and 102.39 ± 2.21 (bilateral) days.

### 3.3. Safety

#### 3.3.1. SAEs and Deaths

SAEs were reported in 7 (4.5%) patients with unilateral DME and 22 (4.8%) patients with bilateral DME. One case of ocular hypertension was classified as an SAE that was not suspected to be related to ranibizumab (Table 3). Cardiac disorders were the most frequently observed SAEs in unilateral (2 (1.3%)) and bilateral (6 (1.3%)) patients with DME. Ischemic stroke, hypertension, and ischemic cardiomyopathy (1 each, 0.2%) in bilateral DME were suspected to be related to the study drug. One (0.6%) patient with unilateral DME and 2 (0.4%) patients with bilateral DME died during the study due to acute renal failure, lung carcinoma, and cardiac arrest; none of them were considered to be related to ranibizumab treatment.

#### 3.3.2. Adverse Events

The occurrence of one or more AEs was reported in 12 (7.6%) patients with unilateral DME and 49 (10.8%) patients with bilateral DME. The percentages of patients with at least one ocular AE were 5 (3.2%) and 19 (4.2%) among the unilateral and bilateral DME patients. The proportions of patients with systemic AEs were 7 (4.5%) and 36 (8.0%) in unilateral and bilateral patients, respectively.

Among patients with unilateral and bilateral DME, eye disorders (5 (3.2%) and 19 (4.2%)), cardiac disorders (2 (1.3%) and 6 (1.3%)), and nervous system disorders (2 (1.3%) and 5 (1.1%)) were the most frequently observed SOC. The nonocular AEs of infections and infestations were observed only in patients with bilateral DME (8 (1.8%); Table 4).

As assessed by the investigator, the AEs suspected to be related to ranibizumab treatment were reported in 1 (0.6%) patient with unilateral DME and 7 (1.5%) patients with bilateral DME. In the patient with unilateral DME, these AEs were categorized as ocular hypertension and cataract. In patients with bilateral DME, 3 patients reported eye disorders suspected to be related to the study drug, 2 categorized with the cataract (0.4%), and the other one with increased lacrimation (0.2%).

The other AEs possibly related to study drug were ischemic stroke (SAE), hypertension (SAE), ischemic cardiomyopathy (SAE), microalbuminuria, and IOP increase.

#### 3.3.3. AEs Leading to Discontinuation

Eight patients (5.1%) with unilateral DME and 14 (3.1%) patients with bilateral DME discontinued the study due to AEs. In patients with unilateral and bilateral DME, cardiac disorders (2 (1.3%) and 4 (0.9%)) and nervous system disorders (2 (1.3%) and 3 (0.7%)) were the most frequent AEs leading to discontinuation. Two (0.4%) patients with bilateral DME and 1 (0.6%) patient with unilateral DME discontinued for events categorized as vascular disorders (arterial disorder in unilateral DME and hypertension and hypertensive crisis in bilateral DME).

### 3.4. Efficacy

**Figure 2:**
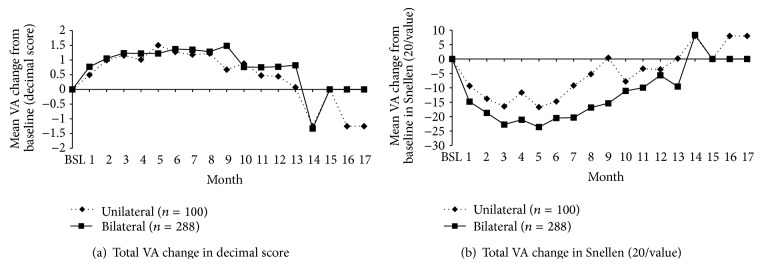
Total VA change (treated eye) from baseline during the 18-month follow-up period in (a) decimal score and in (b) Snellen 20/value, in both unilateral and bilateral patients with DME (per protocol population). Note: the VA loss (in decimal and Snellen scores) at Month 14 was due to treatment interruptions at Month 12 and Month 13 because of ocular stability. Patients were again treated at Month 14 and this led to an increase in VA response at Month 15; BSL, baseline; DME, diabetic macular edema; VA, visual acuity.

Patients recruited in the study had no therapeutic alternative; over 90% of the patients received mainly laser and/or bevacizumab prior to study enrollment and, hence, were not treatment-naïve. Overall, treatment with ranibizumab helped either improve or maintain VA of the treated eye in patients with both unilateral and bilateral DME. The mean baseline VA (in Snellen (20/value)/decimal score) was 73.17/4.11 and 88.02/3.72 in patients with unilateral and bilateral DME, respectively. The Snellen fraction denominators of total VA measured on the treated eye tended to decrease during the study in unilateral and bilateral DME patients, by a mean (± SD) of −16.7 ± 37.84 and −23.62 ± 40.51 Snellen (20/value) compared with baseline VA. In terms of decimal score, the mean (±SD) VA gain in unilateral and bilateral patients was 1.5 ± 2.38 and 1.22 ± 1.67 at Month 5, respectively, compared with baseline VA (Figure 2). The VA loss (in decimal and Snellen scores) at Month 14 was due to treatment interruptions at Month 12 and Month 13 because of ocular stability (the patient's VA was stable for two consecutive monthly assessments). Patients were treated again at Month 14 which led to an increase in VA response at Month 15.

#### 3.4.1. Proportion of Patients with Improvement, Worsening, or No Change in VA at Each Visit in the First 6 Months of Follow-Up

**Figure 3:**
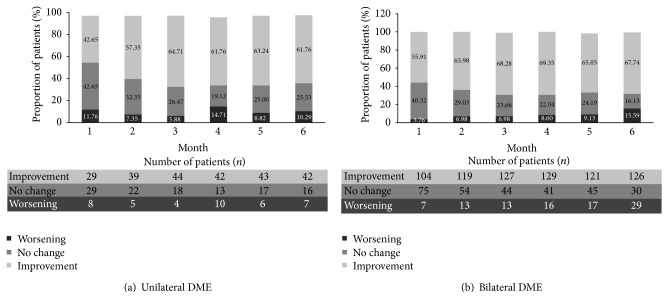
Proportion of patients with total VA (Snellen) improvement, no change, or worsening in patients with at least 6 months of follow-up, in both (a) unilateral and (b) bilateral patients with DME. “Improvement” was defined as a decrease in the total visual acuity fraction denominator at considered visit versus baseline, “no change” as no difference in the total visual acuity fraction denominator at considered visit versus baseline, and “worsening” as an increase in the total visual acuity fraction denominator at considered visit versus baseline. DME, diabetic macular edema; VA, visual acuity.

The percentage of “improvement” in VA through visits was found to be higher than that of “worsening” and “no change” for both unilateral (61.2% (improvement) versus 10.2% (worsening) and 24.5% (no change)) and bilateral patients (68.8% (improvement) versus 16.8% (worsening) and 13.6% (no change)) with DME. The proportion of unilateral patients with a total VA improvement increased from 40.2% at Month 1 to 61.2% over 6 months of follow-up. Similarly, in bilateral patients with DME, the proportion increased from 52.5% to 68.8% from Month 1 to Month 6 (Figure 3).

### 3.5. Subgroups

#### 3.5.1. Patients Who Were Monocular or with BCVA <20/320 Snellen in the Fellow Eye

A decrease in the Snellen fraction denominators and a gain in the decimal scores were observed in both unilateral and bilateral patients (up to −27.4 and 0.41 at visit 3 (Month 1) and up to −50.6 and 2.39 at visit 6 (Month 4), resp., considering only the first visits involved the maximum number of patients) when compared with mean baseline VA (in Snellen (20/value)/decimal score) of 132.09/3.12 and 189.45/2.99 of unilateral and bilateral patients with DME.

#### 3.5.2. Efficacy Outcome in Patients with at Least 6 Months of Follow-Up

**Figure 4:**
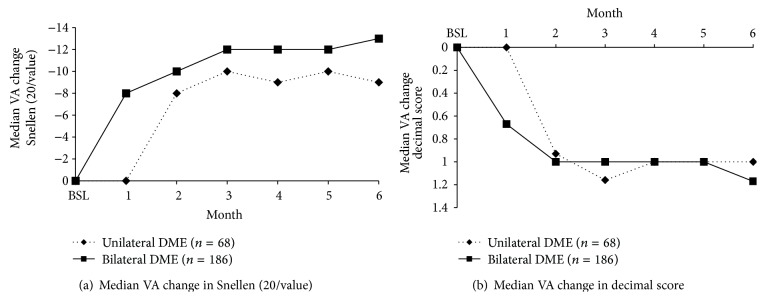
Median VA change in the treated eye from baseline in (a) Snellen (20/value) and (b) decimal score in the subgroup with at least 6 months of follow-up in unilateral and bilateral patients with DME. BSL, baseline; DME, diabetic macular edema; VA, visual acuity.

A total of 254 patients had at least 6 months of follow-up. Greatest improvement in the median VA change was observed after 3-4 months from the start of the study, with a decrease in denominators equal to −9 (unilateral) and −12 (bilateral; Figure 4) in terms of Snellen (20/value) and a gain of 1 decimal score in both unilateral and bilateral patients with DME. The Wilcoxon signed ranks tests showed that the total VA after 6 months of follow-up reached a strong statistical significance (*p* < 0.0001) for both unilateral and bilateral patients with DME compared with baseline (Figure 4).

## 4. Discussion

The PRIDE study evaluated the outcome of an EAP in Italy that aimed to provide early access to ranibizumab treatment for patients with DME who had no therapeutic alternative; most patients were not naïve to treatment before entering the study (i.e., received several different treatments including laser, ranibizumab, and/or bevacizumab). As a result of this study, 612 patients with DME received ranibizumab treatment, while reimbursement was under consideration by the Italian Medicines Agency (Agenzia Italiana del Farmaco). After reimbursement was granted on December 6, 2012, cancellation of the PRIDE EAP study resulted in a variable follow-up period ranging from a minimum of 1 month to a maximum of 18 months for enrolled patients, with the majority completing 6 months of follow-up in the study. The PRIDE study recruited outpatients from 33 centers in Italy and was largely reflective of the real-world clinical setting. Overall, ranibizumab was well-tolerated in the Italian patient population, with no new ocular or nonocular safety findings during EAP. Ranibizumab treatment either improved or maintained VA in both unilateral and bilateral patients with DME.

The incidence of ocular and nonocular AEs and SAEs during the study period was low and consistent with those observed in randomized clinical trials of ranibizumab 0.5 mg PRN in patients with visual impairment due to DME [11, 13, 14, 16–18]. One patient with unilateral DME (0.6%) and two with bilateral DME (0.4%) died during the study primarily due to acute renal failure, lung carcinoma, and cardiac arrest. None of the deaths were considered to be related to ranibizumab treatment.

In the PRIDE study, ranibizumab treatment was based on the EU SmPC 2011, driven by VA loss [21]. Most patients received 2–6 injections, with unilateral patients with DME receiving slightly fewer injections than those with bilateral DME. In patients who completed 6 months of the follow-up period, the greatest improvement in median VA change (Snellen (20/value)) was observed after 3-4 months from the start of the study. The average number of injections received by patients in the PRIDE study was comparable to the recent ranibizumab studies in DME, such as RELIGHT [22] (≈8 in follow-up period of 18 months; patients received monthly PRN in first 6 months and bimonthly PRN treatment thereafter) and RETAIN [23] (≈10 in 24 months in the PRN arm) which resulted in a mean change in BCVA of 6.5 letters (PRN arm), in the RELIGHT study, and 7.44 letters (PRN arm), in the RETAIN study. This trend was also observed in the long-term RESTORE and DRCR.net (Protocol I) studies which showed that the initial BCVA improvements observed at Year 1 were maintained through Years 2 and 3 with a reduced number of injections [11, 13, 14, 17, 18].

The PRIDE study which is reflective of real-world data likewise showed that not all patients required monthly treatment. The lower injection numbers and consequent lower VA outcomes in the PRIDE study may be explained by the retreatment criteria used in the study. The current approved EU SmPC 2014 for ranibizumab addresses the need for individualized treatment [21]. Flexibility in monitoring and retreatment allows for a fully personalized treatment approach. This includes injecting every month until VA stabilizes; option to extend monitoring by four weekly intervals, if VA remains stable, for a maximum of 12 weeks; and retreatment based on VA and/or morphological parameters.

Overall, during the EAP, treatment with ranibizumab either improved or maintained VA of the treated eye in both patients with unilateral or bilateral DME. In addition, a subgroup analysis further corroborated that VA either improved or was maintained in the majority of patients in this study. Approximately, 1/2 of the patients demonstrated an initial VA improvement and achieved stability as early as Months 2-3, with 3-4 ranibizumab injections. Patients were treated as per the EU SmPC, 2011, which mandated ranibizumab injections until VA was stable for 3 consecutive months. Categories of total VA change classified as “improvement,” “worsening,” or “no change” also showed better efficacy outcomes with ranibizumab treatment in the treated eyes, and the percentages of improvement through visits were found to be definitively higher than the proportions of “worsening” and “no change” for both unilateral and bilateral patients with DME.

Analyses repeated on subgroups of particular interest, that is, patients who were monocular or with BCVA <20/320 Snellen in the fellow eye and patients who had completed 6 months of the follow-up period, confirmed the evidence emerging from the safety and the per-protocol populations. Ranibizumab injections were associated on average with VA improvement in the treated eye that reached statistical significance during 6 months of the follow-up period.

The PRIDE study had certain limitations. About 3% of the enrolled patients received laser treatment in the study eye that was reported as a protocol deviation in the study. Overall, 9% of the enrolled patients had ocular disorders in the study eye that could confound the interpretation of results, compromise VA, or required medical or surgical intervention during the study period (reported as protocol deviation), possibly influencing the VA outcomes. Another limitation of the study was that the effect of pretreatment with laser (reported as protocol deviation) or anti-VEGFs including bevacizumab or ranibizumab that may have an impact on the overall BCVA outcomes of such patients was not analyzed separately during the course of the study. Also, caution needs to be exercised when comparing outcomes of PRIDE (real-world experience), with the randomized clinical trials investigating ranibizumab for visual impairment due to DME.

The PRIDE study, an EAP, demonstrated that ranibizumab administered according to the EU SmPC, 2011 was generally well-tolerated and either improved or maintained VA in patients with DME, despite the fact that more than 90% of the patients were not treatment-naïve. The current study adds to the overall real-world clinical experience of ranibizumab in patients with visual impairment due to DME.

## Figures and Tables

**Table 1:** Baseline demographic and disease characteristics.

Characteristics	Unilateral DME (*n* = 157)	Bilateral DME (*n* = 455)
Age, mean ± SD, years	66.36 ± 9.1	64.69 ± 9.2
Male (*n*%)	60.51	63.74
Caucasians (*n*%)	100	99.34
Baseline BCVA, mean ± SD		
Decimal score (value/10)	3.98 ± 2.24	3.56 ± 2.03
Snellen (20/value)	82.13 ± 84.71	91.95 ± 142.00
Age at first DME diagnosis, mean ± SD, years	63.84 ± 9.34	61.65 ± 9.23
Time from DME diagnosis to study entry, mean ± SD, years	2.57 ± 3.35	3.26 ± 3.11

BCVA, best-corrected visual acuity; DME, diabetic macular edema; SD, standard deviation.

**Table 2:** Number of ranibizumab treatments received during the 18-month follow-up period.

	Unilateral DME (*n* = 157)	Bilateral DME (*n* = 455)
Number of injections, mean ± SD	4.15 ± 1.99	4.40 ± 2.11
Number of visits, mean ± SD	7.15 ± 3.57	7.09 ± 3.41
Number of injections/number of visits ratio, mean ± SD	0.62 ± 0.18	0.65 ± 0.19

DME, diabetic macular edema; SD, standard deviation.

**Table 3:** Incidences of SAEs during the 18-month follow-up period (safety set).

Preferred term, *n* (%)	Unilateral DME (*n* = 157)	Bilateral DME (*n* = 455)
Any SAE	**7 (4.46)**	**22 (4.84)**
Cardiac disorders	2 (1.27)	6 (1.32)
Acute myocardial infarction	0	1 (0.22)
Atrial flutter	1 (0.64)	0
Cardiac arrest	0	1 (0.22)
Cardiac failure	0	3 (0.66)
Cardiopulmonary failure	0	1 (0.22)
Congestive cardiomyopathy	1 (0.64)	0
Ischemic cardiomyopathy	0	1 (0.22)
Myocardial ischemia	1 (0.64)	0
Eye disorders	0	2 (0.44)
General disorders and administration site conditions	0	1 (0.22)
Infections and infestations	0	3 (0.66)
Injury, poisoning, and procedural complications	0	2 (0.44)
Metabolism and nutrition disorders	0	1 (0.22)
Musculoskeletal and connective tissue disorders	0	1 (0.22)
Neoplasms benign, malignant, and unspecified (including cysts and polyps)	1 (0.64)	3 (0.66)
Nervous system disorders	2 (1.27)	3 (0.66)
Renal and urinary disorders	1 (0.64)	1 (0.22)
Respiratory, thoracic, and mediastinal disorders	1 (0.64)	1 (0.22)
Surgical and medical procedures	0	1 (0.22)
Vascular disorders	1 (0.64)	1 (0.22)

DME, diabetic macular edema; SAE, serious adverse event.

**Table 4:** Incidences of AEs during the 18-month follow-up period (safety set).

Preferred term, *n* (%)	Unilateral DME (*n* = 157)	Bilateral DME (*n* = 455)
Any AE	**12 (7.64)**	**49 (10.77)**
Blood and lymphatic system disorders	0	1 (0.22)
Cardiac disorders	2 (1.27)	6 (1.32)
Eye disorders (mainly cataract, conjunctival hemorrhage, and diabetic retinal edema being the major)	5 (3.18)	19 (4.18)
General disorders and administration site conditions	0	2 (0.44)
Infections and infestations	0	8 (1.76)
Injury, poisoning, and procedural complications	0	3 (0.66)
Investigations	0	1 (0.22)
Metabolism and nutrition disorders	0	1 (0.22)
Musculoskeletal and connective tissue disorders	0	1 (0.22)
Neoplasms benign, malignant, and unspecified (including cysts and polyps)	1 (0.64)	4 (0.88)
Nervous system disorders	2 (1.27)	5 (1.10)
Psychiatric disorders	0	1 (0.22)
Renal and urinary disorders	1 (0.64)	2 (0.44)
Respiratory, thoracic, and mediastinal disorders	1 (0.64)	1 (0.22)
Skin and subcutaneous tissue disorders	0	1 (0.22)
Surgical and medical procedures	0	4 (0.88)
Vascular disorders	1 (0.64)	3 (0.66)

AE, adverse event; DME, diabetic macular edema.
